# Supplementary material for: Dynalign II: common secondary structure prediction for RNA homologs with domain insertions
Source: Nucleic Acids Res. 2014 Nov 21;42(22):13939–48. doi: 10.1093/nar/gku1172 (PMC4267632; doi:10.1093/nar/gku1172)
Supplement: SUPPLEMENTARY DATA [file supp_gku1172_nar-02021-z-2014-File012.zip › manual/javadoc/RNAstructure_java_interface/source/windows/InternalWindow.html]

InternalWindow


JavaScript is disabled on your browser.


- Overview
- Package
- Class
- Tree
- Index
- Help

- Prev Class
- Next Class

- Frames
- No Frames

- All Classes

- Summary:
- Nested |
- Field |
- Constr |
- Method

- Detail:
- Field |
- Constr |
- Method


RNAstructure\_java\_interface.source.windows

## Class InternalWindow

- java.lang.Object
- - java.awt.Component
  - - java.awt.Container
    - - javax.swing.JComponent
      - - javax.swing.JInternalFrame
        - - RNAstructure\_java\_interface.source.windows.InternalWindow

- All Implemented Interfaces:
  :   ActionListener, ImageObserver, MenuContainer, Serializable, EventListener, Accessible, RootPaneContainer, WindowConstants

  Direct Known Subclasses:
  :   DrawingWindow, ModuleWindow, SequenceDisplayWindow

  ---

    

  ```
  public abstract class InternalWindow
  extends JInternalFrame
  implements ActionListener
  ```

  A class that holds internal frames for the RNAstructure GUI.
    
    
  Every frame is connected to the back end through an individual proxy.

  See Also:
  :   Serialized Form

- - ### Nested Class Summary

    - ### Nested classes/interfaces inherited from class javax.swing.JInternalFrame

      `JInternalFrame.AccessibleJInternalFrame, JInternalFrame.JDesktopIcon`
    - ### Nested classes/interfaces inherited from class javax.swing.JComponent

      `JComponent.AccessibleJComponent`
    - ### Nested classes/interfaces inherited from class java.awt.Container

      `Container.AccessibleAWTContainer`
    - ### Nested classes/interfaces inherited from class java.awt.Component

      `Component.AccessibleAWTComponent, Component.BaselineResizeBehavior, Component.BltBufferStrategy, Component.FlipBufferStrategy`
  - ### Field Summary

    Fields

    | Modifier and Type | Field and Description |
    | --- | --- |
    | `protected RNAstructureBackendCalculator` | `backend` The back end calculator that's linked to this window. |
    | `protected SimpleDialogHandler` | `dialogHandler` A dialog handler to show any simple dialog messages that pop up from this window. |
    | `protected DynamicMenuBar` | `menuBar` The menu bar that's attached to this window. |
    | `private static long` | `serialVersionUID` |

    - ### Fields inherited from class javax.swing.JInternalFrame

      `closable, CONTENT_PANE_PROPERTY, desktopIcon, FRAME_ICON_PROPERTY, frameIcon, GLASS_PANE_PROPERTY, iconable, IS_CLOSED_PROPERTY, IS_ICON_PROPERTY, IS_MAXIMUM_PROPERTY, IS_SELECTED_PROPERTY, isClosed, isIcon, isMaximum, isSelected, LAYERED_PANE_PROPERTY, maximizable, MENU_BAR_PROPERTY, resizable, ROOT_PANE_PROPERTY, rootPane, rootPaneCheckingEnabled, title, TITLE_PROPERTY`
    - ### Fields inherited from class javax.swing.JComponent

      `accessibleContext, listenerList, TOOL_TIP_TEXT_KEY, ui, UNDEFINED_CONDITION, WHEN_ANCESTOR_OF_FOCUSED_COMPONENT, WHEN_FOCUSED, WHEN_IN_FOCUSED_WINDOW`
    - ### Fields inherited from class java.awt.Component

      `BOTTOM_ALIGNMENT, CENTER_ALIGNMENT, LEFT_ALIGNMENT, RIGHT_ALIGNMENT, TOP_ALIGNMENT`
    - ### Fields inherited from interface javax.swing.WindowConstants

      `DISPOSE_ON_CLOSE, DO_NOTHING_ON_CLOSE, EXIT_ON_CLOSE, HIDE_ON_CLOSE`
    - ### Fields inherited from interface java.awt.image.ImageObserver

      `ABORT, ALLBITS, ERROR, FRAMEBITS, HEIGHT, PROPERTIES, SOMEBITS, WIDTH`
  - ### Constructor Summary

    Constructors

    | Modifier | Constructor and Description |
    | --- | --- |
    | `protected` | `InternalWindow()` Constructor. |
  - ### Method Summary

    Methods

    | Modifier and Type | Method and Description |
    | --- | --- |
    | `void` | `actionPerformed(ActionEvent e)` |
    | `protected abstract void` | `doActions(String command)` Do any actions specific to this window. |
    | `protected abstract RolloverMenu[]` | `setMenus()` Create the array of variable menus used by this window. |
    | `protected void` | `setTitles(String title)` Set the title of both this window and the main window. |
    | `void` | `viewWindow()` View the window. |

    - ### Methods inherited from class javax.swing.JInternalFrame

      `addImpl, addInternalFrameListener, createRootPane, dispose, doDefaultCloseAction, fireInternalFrameEvent, getAccessibleContext, getContentPane, getDefaultCloseOperation, getDesktopIcon, getDesktopPane, getFocusCycleRootAncestor, getFocusOwner, getFrameIcon, getGlassPane, getInternalFrameListeners, getJMenuBar, getLastCursor, getLayer, getLayeredPane, getMenuBar, getMostRecentFocusOwner, getNormalBounds, getRootPane, getTitle, getUI, getUIClassID, getWarningString, hide, isClosable, isClosed, isFocusCycleRoot, isIcon, isIconifiable, isMaximizable, isMaximum, isResizable, isRootPaneCheckingEnabled, isSelected, moveToBack, moveToFront, pack, paintComponent, paramString, remove, removeInternalFrameListener, reshape, restoreSubcomponentFocus, setClosable, setClosed, setContentPane, setCursor, setDefaultCloseOperation, setDesktopIcon, setFocusCycleRoot, setFrameIcon, setGlassPane, setIcon, setIconifiable, setJMenuBar, setLayer, setLayer, setLayeredPane, setLayout, setMaximizable, setMaximum, setMenuBar, setNormalBounds, setResizable, setRootPane, setRootPaneCheckingEnabled, setSelected, setTitle, setUI, show, toBack, toFront, updateUI`
    - ### Methods inherited from class javax.swing.JComponent

      `addAncestorListener, addNotify, addVetoableChangeListener, computeVisibleRect, contains, createToolTip, disable, enable, firePropertyChange, firePropertyChange, firePropertyChange, fireVetoableChange, getActionForKeyStroke, getActionMap, getAlignmentX, getAlignmentY, getAncestorListeners, getAutoscrolls, getBaseline, getBaselineResizeBehavior, getBorder, getBounds, getClientProperty, getComponentGraphics, getComponentPopupMenu, getConditionForKeyStroke, getDebugGraphicsOptions, getDefaultLocale, getFontMetrics, getGraphics, getHeight, getInheritsPopupMenu, getInputMap, getInputMap, getInputVerifier, getInsets, getInsets, getListeners, getLocation, getMaximumSize, getMinimumSize, getNextFocusableComponent, getPopupLocation, getPreferredSize, getRegisteredKeyStrokes, getSize, getToolTipLocation, getToolTipText, getToolTipText, getTopLevelAncestor, getTransferHandler, getVerifyInputWhenFocusTarget, getVetoableChangeListeners, getVisibleRect, getWidth, getX, getY, grabFocus, isDoubleBuffered, isLightweightComponent, isManagingFocus, isOpaque, isOptimizedDrawingEnabled, isPaintingForPrint, isPaintingOrigin, isPaintingTile, isRequestFocusEnabled, isValidateRoot, paint, paintBorder, paintChildren, paintImmediately, paintImmediately, print, printAll, printBorder, printChildren, printComponent, processComponentKeyEvent, processKeyBinding, processKeyEvent, processMouseEvent, processMouseMotionEvent, putClientProperty, registerKeyboardAction, registerKeyboardAction, removeAncestorListener, removeNotify, removeVetoableChangeListener, repaint, repaint, requestDefaultFocus, requestFocus, requestFocus, requestFocusInWindow, requestFocusInWindow, resetKeyboardActions, revalidate, scrollRectToVisible, setActionMap, setAlignmentX, setAlignmentY, setAutoscrolls, setBackground, setBorder, setComponentPopupMenu, setDebugGraphicsOptions, setDefaultLocale, setDoubleBuffered, setEnabled, setFocusTraversalKeys, setFont, setForeground, setInheritsPopupMenu, setInputMap, setInputVerifier, setMaximumSize, setMinimumSize, setNextFocusableComponent, setOpaque, setPreferredSize, setRequestFocusEnabled, setToolTipText, setTransferHandler, setUI, setVerifyInputWhenFocusTarget, setVisible, unregisterKeyboardAction, update`
    - ### Methods inherited from class java.awt.Container

      `add, add, add, add, add, addContainerListener, addPropertyChangeListener, addPropertyChangeListener, applyComponentOrientation, areFocusTraversalKeysSet, countComponents, deliverEvent, doLayout, findComponentAt, findComponentAt, getComponent, getComponentAt, getComponentAt, getComponentCount, getComponents, getComponentZOrder, getContainerListeners, getFocusTraversalKeys, getFocusTraversalPolicy, getLayout, getMousePosition, insets, invalidate, isAncestorOf, isFocusCycleRoot, isFocusTraversalPolicyProvider, isFocusTraversalPolicySet, layout, list, list, locate, minimumSize, paintComponents, preferredSize, printComponents, processContainerEvent, processEvent, remove, removeAll, removeContainerListener, setComponentZOrder, setFocusTraversalPolicy, setFocusTraversalPolicyProvider, transferFocusDownCycle, validate, validateTree`
    - ### Methods inherited from class java.awt.Component

      `action, add, addComponentListener, addFocusListener, addHierarchyBoundsListener, addHierarchyListener, addInputMethodListener, addKeyListener, addMouseListener, addMouseMotionListener, addMouseWheelListener, bounds, checkImage, checkImage, coalesceEvents, contains, createImage, createImage, createVolatileImage, createVolatileImage, disableEvents, dispatchEvent, enable, enableEvents, enableInputMethods, firePropertyChange, firePropertyChange, firePropertyChange, firePropertyChange, firePropertyChange, firePropertyChange, getBackground, getBounds, getColorModel, getComponentListeners, getComponentOrientation, getCursor, getDropTarget, getFocusListeners, getFocusTraversalKeysEnabled, getFont, getForeground, getGraphicsConfiguration, getHierarchyBoundsListeners, getHierarchyListeners, getIgnoreRepaint, getInputContext, getInputMethodListeners, getInputMethodRequests, getKeyListeners, getLocale, getLocation, getLocationOnScreen, getMouseListeners, getMouseMotionListeners, getMousePosition, getMouseWheelListeners, getName, getParent, getPeer, getPropertyChangeListeners, getPropertyChangeListeners, getSize, getToolkit, getTreeLock, gotFocus, handleEvent, hasFocus, imageUpdate, inside, isBackgroundSet, isCursorSet, isDisplayable, isEnabled, isFocusable, isFocusOwner, isFocusTraversable, isFontSet, isForegroundSet, isLightweight, isMaximumSizeSet, isMinimumSizeSet, isPreferredSizeSet, isShowing, isValid, isVisible, keyDown, keyUp, list, list, list, location, lostFocus, mouseDown, mouseDrag, mouseEnter, mouseExit, mouseMove, mouseUp, move, nextFocus, paintAll, postEvent, prepareImage, prepareImage, processComponentEvent, processFocusEvent, processHierarchyBoundsEvent, processHierarchyEvent, processInputMethodEvent, processMouseWheelEvent, remove, removeComponentListener, removeFocusListener, removeHierarchyBoundsListener, removeHierarchyListener, removeInputMethodListener, removeKeyListener, removeMouseListener, removeMouseMotionListener, removeMouseWheelListener, removePropertyChangeListener, removePropertyChangeListener, repaint, repaint, repaint, resize, resize, setBounds, setBounds, setComponentOrientation, setDropTarget, setFocusable, setFocusTraversalKeysEnabled, setIgnoreRepaint, setLocale, setLocation, setLocation, setName, setSize, setSize, show, size, toString, transferFocus, transferFocusBackward, transferFocusUpCycle`
    - ### Methods inherited from class java.lang.Object

      `clone, equals, finalize, getClass, hashCode, notify, notifyAll, wait, wait, wait`

- - ### Field Detail


    - #### backend

      ```
      protected final RNAstructureBackendCalculator backend
      ```

      The back end calculator that's linked to this window.


    - #### dialogHandler

      ```
      protected final SimpleDialogHandler dialogHandler
      ```

      A dialog handler to show any simple dialog messages that pop up from
      this window.


    - #### menuBar

      ```
      protected DynamicMenuBar menuBar
      ```

      The menu bar that's attached to this window.


    - #### serialVersionUID

      ```
      private static final long serialVersionUID
      ```

      See Also:
      :   Constant Field Values
  - ### Constructor Detail


    - #### InternalWindow

      ```
      protected InternalWindow()
      ```

      Constructor.
  - ### Method Detail


    - #### actionPerformed

      ```
      public void actionPerformed(ActionEvent e)
      ```

      **Specified by:**
      :   `actionPerformed` in interface `ActionListener`


    - #### doActions

      ```
      protected abstract void doActions(String command)
      ```

      Do any actions specific to this window.

      Parameters:
      :   `command` - The command that signifies a particular action.


    - #### setMenus

      ```
      protected abstract RolloverMenu[] setMenus()
      ```

      Create the array of variable menus used by this window.

      Returns:
      :   The array of variable menus created for this window.


    - #### setTitles

      ```
      protected void setTitles(String title)
      ```

      Set the title of both this window and the main window.

      Parameters:
      :   `title` - The new title.


    - #### viewWindow

      ```
      public void viewWindow()
      ```

      View the window.


- Overview
- Package
- Class
- Tree
- Index
- Help

- Prev Class
- Next Class

- Frames
- No Frames

- All Classes

- Summary:
- Nested |
- Field |
- Constr |
- Method

- Detail:
- Field |
- Constr |
- Method
